# Exploring the alleviating effects of *Bifidobacterium* metabolite lactic acid on non-alcoholic steatohepatitis through the gut-liver axis

**DOI:** 10.3389/fmicb.2024.1518150

**Published:** 2025-01-07

**Authors:** Hongmei Zhao, Juan Zhou, Lingzhi Yuan, Zhiyi Sun, Yi Liu, Xinyu Zhao, Feng Ye

**Affiliations:** ^1^Department of Infectious Disease, The First Affiliated Hospital of Xi'an Jiaotong University, Xi'an, China; ^2^Department of Gastroenterology and Nutrition, The Affiliated Children's Hospital of Xiangya School of Medicine, Central South University (Hunan Children's Hospital), Changsha, China; ^3^University of Michigan Medical School, Ann Arbor, MI, United States; ^4^Department of Traditional Chinese Medicine, The First Affiliated Hospital of Xi'an Jiaotong University, Xi'an, China; ^5^Department of Pediatrics, Changsha County Maternal and Child Health Hospital, Changsha, China

**Keywords:** NAFLD, *Bifidobacterium*, gut-liver axis, lactic acid, NLRP3 inflammasome, autophagy

## Abstract

**Objective:**

This study investigates the protective effects of lactic acid, a metabolite of *Bifidobacterium*, on non-alcoholic fatty liver disease (NAFLD) induced by a high-sugar, high-fat diet (HFD) in mice, in the context of the gut-liver axis.

**Methods:**

A NAFLD mouse model was established using a HFD, and different intervention groups were set up to study the protective effects of *Bifidobacterium* and its metabolite lactic acid. The groups included a control group, NAFLD group, *Bifidobacterium* treatment group, Glyceraldehyde-3-P (G-3P) co-treatment group, and NOD-like receptor family pyrin domain containing 3 (NLRP3) overexpression group. The evaluation of liver function and lipid metabolism was conducted using the liver-to-body weight ratio, histological staining, and biochemical assays. Enzyme-linked immunosorbent assay (ELISA) was performed to measure inflammatory cytokines, and western blotting was used to analyze the expression of NLRP3 inflammasome and autophagy-related molecules. *In vitro*, an NAFLD cell model was established using oleic acid, with cells treated with lactic acid and NLRP3 overexpression to assess lipid droplet accumulation and inflammation.

**Results:**

*In vivo* findings indicated that, in comparison to CBX group (Control group without antibiotic treatment), NAFLD/CBX group (NAFLD group without antibiotic administration) and NAFLD/ABX group (NAFLD group with antibiotic administration) exhibited increased liver-to-body weight ratio, higher lipid droplet accumulation, aggravated liver histopathological damage, and elevated levels of AST (Aspartate Aminotransferase), ALT (Alanine Aminotransferase), TC (Total Cholesterol), TG (Triglycerides), LDL-C (Low-Density Lipoprotein Cholesterol), IL-6 (Interleukin-6), TNF-α (Tumor Necrosis Factor-alpha), IL-1β (Interleukin-1 beta), and NLRP3-related molecules, while HDL-C (High-Density Lipoprotein Cholesterol) levels significantly decreased. Intervention with *Bifidobacterium* significantly reversed these adverse changes. Further addition of G-3P led to more pronounced improvement in NAFLD symptoms, while overexpression of NLRP3 weakened the protective effects of *Bifidobacterium*. *In vitro* results indicated that Ole group exhibited heightened lipid droplet accumulation and expression of NLRP3 inflammasome-related molecules relative to the control group. Treatment with lactic acid effectively reversed these changes; however, the protective effect of lactic acid was significantly weakened with NLRP3 overexpression.

**Conclusion:**

Lactic acid can alleviate lipid metabolism disorders in NAFLD induced by diet through the inhibition of inflammation mediated by the NLRP3 inflammasome and the regulation of the autophagy process.

## 1 Introduction

Non-alcoholic fatty liver disease (NAFLD) is the most common chronic liver condition globally, characterized by fat accumulation, inflammation, and hepatocyte injury (Nassir, 2022). Current treatment treatments primarily focus on lifestyle changes and weight reduction, but often show limited effectiveness (Rong et al., 2022). Therefore, understanding the underlying mechanisms of NAFLD is crucial for developing more effective treatments.

The gut microbiota plays a pivotal role in host health, influencing metabolism and immune function (Wastyk et al., 2021). The gut microbiota impacts multiple organs through axes such as the gut-brain, gut-liver, and gut-heart axes (Colella et al., 2023). Notably, the gut-liver axis mechanism the effect of gut microbiota on liver function *via* its metabolites (Vallianou et al., 2021). Disruption of the gut microbiota and its metabolites has been implicated in the onset and progression of NAFLD (Bauer et al., 2022). Dysbiosis and aberrant metabolite production are significant pathogenic factors in NAFLD (Raza et al., 2021). Changes in the gut microbiota can impair the intestinal barrier, leading to the translocation of endotoxins into the bloodstream, which triggers liver inflammation and lipid metabolism disorders, worsening NAFLD (Ruiz de Galarreta et al., 2023; Chausiaux et al., 2022).

Modulating gut microbiota and its metabolites may provide a new approach for managing NAFLD. Bifidobacteria, as an important group of probiotics, have been shown to improve gut microbiota composition, regulate metabolism, and alleviate inflammation (Hao et al., 2021; Yoshimura et al., 2022). Compared to traditional pharmacotherapy, Bifidobacteria offer a natural, safe, and low-side-effect alternative, making them suitable for long-term prevention and treatment (Saez-Lara et al., 2015). Lactic acid, a key metabolic product of Bifidobacteria, regulates gut microbiota balance, inhibits pathogenic bacteria, and modulates immune responses. It has shown potential therapeutic efficacy in NAFLD by enhancing intestinal barrier function and reducing endotoxin levels (Hamad et al., 2022; Mishra et al., 2023). However, the clinical application of Bifidobacteria and their metabolites remains limited due to a lack of understanding of their mechanisms of action. Therefore, further investigation into the specific mechanisms by which Bifidobacteria and lactic acid act in NAFLD is essential.

Dysregulated lipid metabolism is a hallmark of NAFLD, characterized by abnormal lipid (such as fat, cholesterol, and phospholipids) metabolism, leading to lipid accumulation in the liver and tissues (Pawlak et al., 2015). This disruption contributes not only to hepatic fat accumulation but also to the activation of inflammatory responses, exacerbating the severity of NAFLD (Weiss et al., 2023). Studies have shown a strong link between the activation of the NLRP3 inflammasome and the progression of NAFLD (Hu et al., 2020). The NOD-like receptor family pyrin domain containing 3 (NLRP3) inflammasome functions is a key intracellular complex that detects stress signals and triggers inflammation. Overactivation of contributes to hepatic inflammation and injury (Zhang et al., 2022). In NAFLD, excessive NLRP3 activation not only triggers hepatic inflammation but may also inhibit autophagy, aggravating lipid metabolism disorders (Del Campo et al., 2018; Yang et al., 2016). This highlights the need for new interventions to improve NAFLD treatment. Importantly, lactic acid has been shown to inhibit NLRP3 inflammasome activation, suggesting its potential role in alleviating both inflammation and lipid metabolism disorders in NAFLD.

**Figure 1:**
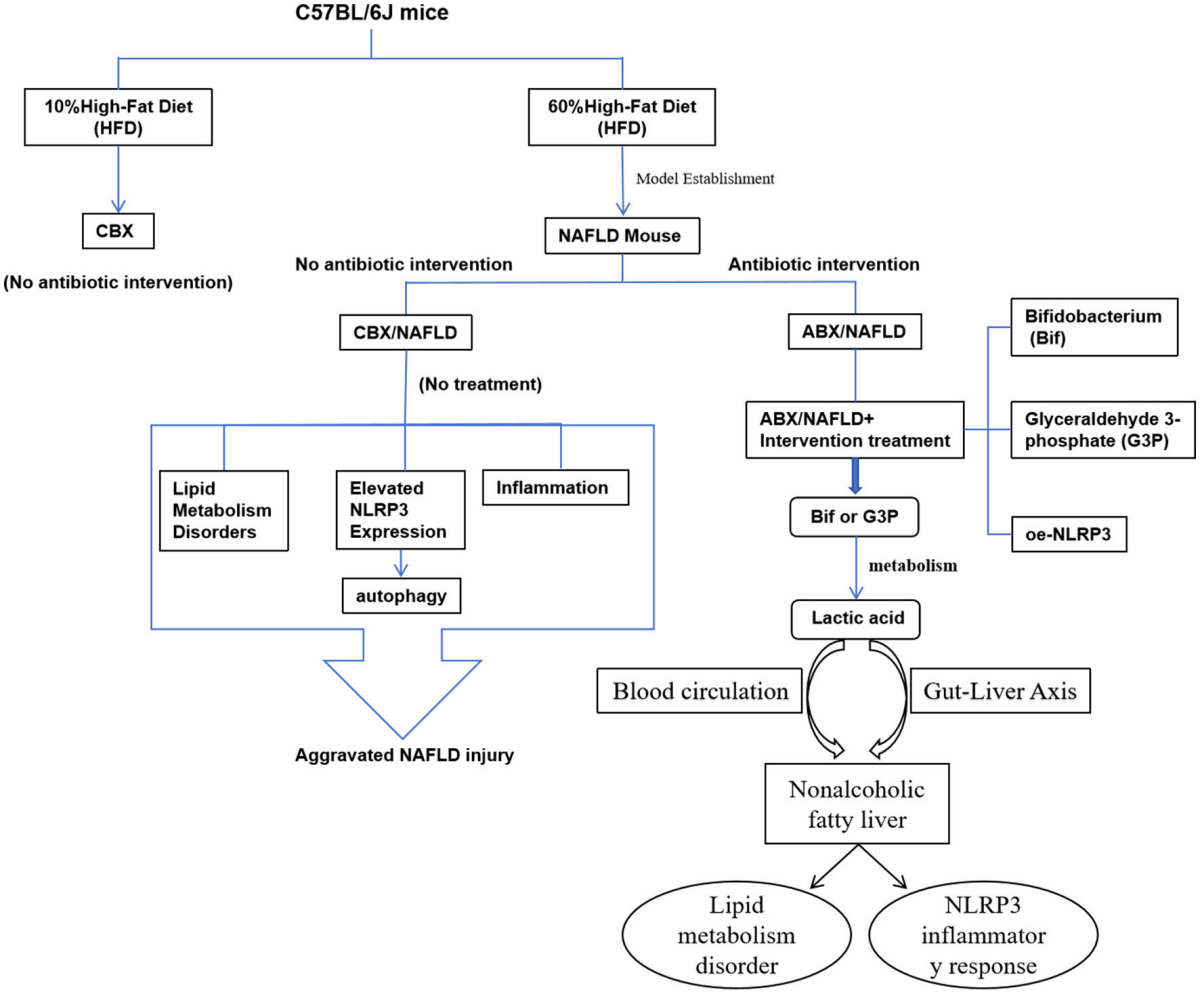
*Bifidobacterium* improves non-alcoholic fatty liver disease through the gut-liver axis metabolism.

In summary, this study aims to explore the mechanisms of the gut-liver axis and investigate the potential role of *Bifidobacterium* (Bif) and its metabolite lactic acid in NAFLD treatment, with the goal of providing new theoretical foundations and effective intervention strategies for NAFLD therapy. Additionally, the findings could guide future clinical treatments, offering more personalized and sustainable therapeutic options for NAFLD patients. The metabolic pathway of *Bifidobacterium* through the gut-liver axis is shown in Figure 1.

## 2 Materials and methods

### 2.1 Animal experiments

Seven-week-old male C57BL/6J mice (Silaike Jingda, Changsha) were housed under standard laboratory conditions (20–26°C, 30%−40% humidity) with a 12-h light/dark cycle, unrestricted access to food and water, and allowed to acclimate for 1 week. The mice were then randomly assigned to 8 groups (*n* = 6 per group, a total of 48 mice):

(1) CBX group (*n* = 6): Administered PBS without antibiotics and fed a 10% high-fat diet (HFD);
(2) NAFLD/CBX group (*n* = 6): Fed a 60% HFD (D12492, Yes Service Biotech, Shanghai);
(3) NAFLD/ABX group (*n* = 6): Mice were administered an antibiotic cocktail (metronidazole 0.2 g/L, vancomycin 0.1 g/L, ampicillin 0.2 g/L, and neomycin 0.2 g/L) *via* gavage for 4 weeks. The antibiotic mixture was filtered through a 0.22 μm membrane before administration, and the mice were concurrently fed a 60% HFD;
(4) NAFLD/ABX+Bif group (Bif) (*n* = 6): Fed a 60% HFD and given 0.2 mL of *Bifidobacterium* daily by gavage for 4 weeks (Li et al., 2022);
(5) NAFLD/ABX+Bif+G3P group (*n* = 6): Fed a 60% HFD and given 0.2 mL of Bif and 300 mg/kg Glyceraldehyde-3-P (G-3-P) daily by gavage for 4 weeks (Simic et al., 2020);
(6) NAFLD/ABX+oe-NLRP3 group (oe-NLRP3) (*n* = 6): Fed a 60% HFD, injected with an NLRP3 overexpression vector *via* the tail vein.
(7) NAFLD/ABX+Bif+oe–NC group (Bif+oe–NC) (*n* = 6): Fed a 60% HFD, given 0.2 mL of Bif daily by gavage, and injected with an empty vector *via* the tail vein;
(8) NAFLD/ABX+Bif+oe–NLRP3 group (Bif+oe–NLRP3) (*n* = 6): Fed a 60% HFD, given 0.2 mL of Bif daily by gavage, and injected with an NLRP3 overexpression vector *via* the tail vein.

Mouse body weight was recorded every 4 days. Mice were then euthanized by cervical dislocation under 2%−3% isoflurane (PHR2874, Merck) anesthesia, and blood, liver, and intestinal tissues were harvested. All experimental procedures were approved by the Institutional Animal Care and Use Committee.

### 2.2 Reagent preparation

The active *Bifidobacterium* preparation (Livzon, Zhuhai, Guangzhou), containing 5 × 107 CFU/mL of *Bifidobacterium* per dose, was dissolved in 0.9% normal saline (NS) to prepare a 0.2 mL suspension for experimental treatment. Approval number: National Medicine Standard Zhun Zi S10960040.

Glyceraldehyde 3-phosphate (G-3-P, G915968, Macklin, Shanghai) was dissolved at a dose of 300 mg/kg in 500 μL of 0.9% normal saline, ensuring thorough mixing before each use.

### 2.3 Oil Red O staining

Liver tissues that had been frozen were cut into slices 6 μm thick and washed with PBS. The sections were stained with Oil Red O staining solution (C0157S, Beyotime, Shanghai) at 4°C for 30 min. After staining, the sections were quickly rinsed with 60% isopropanol to remove excess dye. Cells were washed three times with PBS and fixed in 4% paraformaldehyde (PFA, P0099, Beyotime, Shanghai) for 10 min, followed by two washes with double-distilled water to remove the PFA. The sections were then washed once with 60% isopropanol and stained with Oil Red O again at 37°C for 10 min. After staining, both tissues and cells were quickly rinsed with 60% isopropanol to eliminate any remaining dye. Observations and image capture were performed using an microscope (Olympus, Tokyo, Japan), and image analysis was conducted with Image J software (V1.0.112, NIH, Madison, WI, USA).

### 2.4 Hematoxylin-eosin (HE) staining

Liver tissues were preserved in 4% PFA, embedded in paraffin, and sliced into sections 4 μm thick. After dehydration through a graded series of ethanol (75, 85, 95, and 100%), the sections were stained using HE staining solution (C0105S, Beyotime, Shanghai) and examined with an Olympus microscope (magnification: 100×, 200×) to assess the tissue structure.

### 2.5 Biochemical analysis

Blood samples from mice were collected and centrifuged after standing. Following the manufacturer's guidelines, an automatic analyzer (BS-1000, Mindray, Shenzhen) was employed to measure aspartate aminotransferase (AST, MAK055, Merck), alanine aminotransferase (ALT, MAK052, Merck), total cholesterol (TC, BC1985, Solarbio, Beijing), low-density lipoprotein cholesterol (LDL-C, 60736ES, YESEN, Shanghai), high-density lipoprotein cholesterol (HDL-C, 60736ES, YESEN, Shanghai), and triglycerides (TG, S0219S, Beyotime, Shanghai).

### 2.6 Lactic acid concentration measurement

The concentration of lactic acid in serum, as well as in the contents of the cecum and ileum, was measured using a lactate assay kit (BC2235, Solarbio), according to the manufacturer's instructions.

### 2.7 Construction and grouping of NAFLD cell model

The AML12 mouse hepatocyte cell line (ATCC, Manassas, VA, USA) was maintained in DMEM medium (M150210B, Pricella Biotechnology Co., Ltd., Wuhan) supplemented with 10% fetal bovine serum (FBS) and 1% penicillin/streptomycin at 37°C in a 5% CO_2_ atmosphere. The experimental groups were set as follows: Control (no treatment), Ole (treated with 60 μg/mL oleic acid for 24 h), Ole+Lac (treated with oleic acid and then with 15 mM lactic acid for 15 min), Ole+Lac+oe-NLRP3 (transfected with NLRP3 overexpression plasmid following oleic acid treatment), and Ole+Lac+oe–NC (transfected with empty vector after lactic acid treatment). Transfection of AML12 cells was conducted using Lipofectamine 2000 (11668500, Thermo Fisher, Waltham, MA, USA), with NLRP3-pcDNA3 plasmid (oe-NLRP3, synthesized by SnapGene) as the overexpression vector, and the empty plasmid serving as the negative control (oe-NC).

### 2.8 ELISA

ELISA kits were utilized to quantify the levels of Tumor Necrosis Factor-alpha (TNF-α, PT512, Beyotime, Shanghai), Interleukin-1 beta (IL-1β, PI301, Beyotime, Shanghai), and Interleukin-6 (IL-6, PI326, Beyotime, Shanghai) in cells and tissues, following the instructions provided with the kits.

### 2.9 Western blot

Tissues or cells were lysed with RIPA lysis buffer, and protein concentrations were assessed using a BCA kit. Equal amounts of protein were resolved by 10% SDS-PAGE and subsequently transferred to polyvinylidene fluoride (PVDF) membranes (Millipore). Following a blocking step, the membranes were incubated overnight at 4°C with primary antibodies: NLRP3 (1:1000, ab263899, Abcam, UK), ASC (1:1000, ab309497, Abcam, UK), pro-Casp-1 (1:1000, AB1871, Abclonal, Wuhan), Casp-1 (1:1000, A18646, Abclonal, Wuhan), LC3-II/I (1:1000, A6742, Abclonal, Wuhan), Beclin-1 (1:1000, ab302669, Abcam, UK), pro-IL-1β (1:1000, ab18955, Abcam, UK), IL-1β (1:1000, A16288, Abclonal, Wuhan), and GAPDH (1:1000, ab8245, Abcam, UK). After washing with TBST, membranes were treated with HRP-conjugated secondary antibodies at room temperature for 2 h, followed by additional TBST washes. Chemiluminescent detection was performed using the enhanced chemiluminescence detection kit (P0018S, Beyotime, Shanghai, China), and Image J software (NIH) was utilized for image analysis.

### 2.10 qRT-PCR

Total RNA was extracted using the RNA Extraction Kit (B511311, Sangon Biotech, Shanghai, China), and reverse transcription was performed with PrimeScript™ RT Master Mix (Takara, Kyoto, Japan). Quantitative PCR (qPCR) analysis was conducted using Power SYBR Green PCR Master Mix (Applied Biosystems, CA, USA). Gene expression levels were quantified by the 2^−ΔΔCt^ method, with GAPDH used as an internal control. Primer sequences for the target genes were as follows: NLRP3 (forward: CCCCTTTATTTGTACCCAAGGC, reverse: ATCCCAGCAAACCCATCCAC), ASC (forward: ACAGTACCAGGCAGTTCGTG, reverse: GGTGCCTTTCTAAGCCCCAT), Casp-1 (forward: ACTGCTATGGACAAGGCACG, reverse: GCAAGACGTGTACGAGTGGT), IL-1β (forward: TGCCACCTTTTGACAGTGATG, reverse: TGGGTGTGCCGTCTTTCATT), LC3 (forward: CATGGTCTACGCCTCCCAAG, reverse: CCCAAAAGAGCAACCCGAAC), Beclin-1 (forward: GCTGTAGCCAGCCTCTGAAA, reverse: AATGGCTCCTGTGAGTTCCTG), GAPDH (forward: CCCTTAAGAGGGATGCTGCC, reverse: TACGGCCAAATCCGTTCACA).

### 2.11 Statistical analysis

Statistical analysis was performed using GraphPad Prism 9 (Dotmatics, Boston, MA, USA). Data are presented as mean ± SD. To compare two groups, *t*-tests were employed, whereas for multiple groups, one-way analysis of variance (ANOVA) followed by Bonferroni *post-hoc* tests was conducted. *P* < 0.05 was regarded as indicative of statistical significance.

## 3 Results

### 3.1 *Bifidobacterium* metabolites alleviate lipid metabolism disorders and inflammatory responses in mice

**Figure 2:**
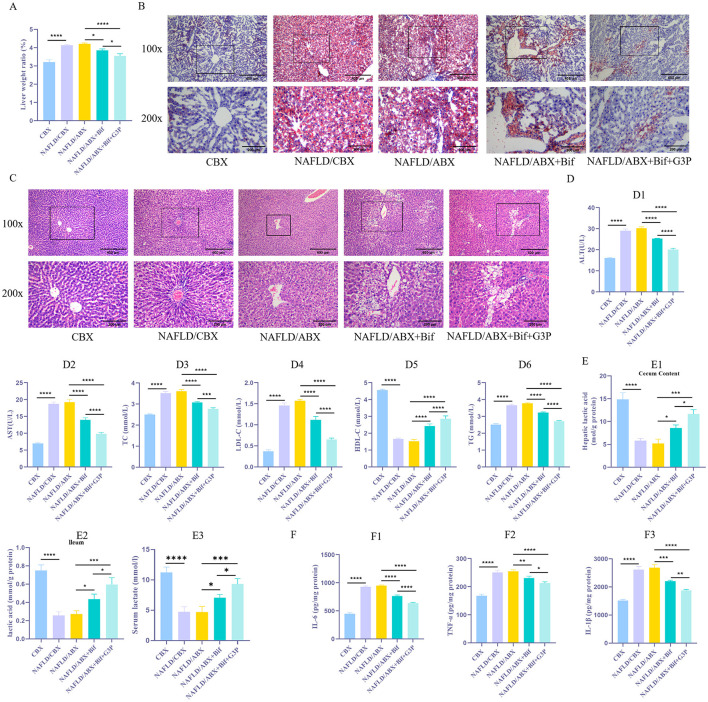
*Bifidobacterium* metabolites alleviate lipid metabolism disorders and inflammatory responses in mice. **(A)** Measurement of liver-to-body weight ratio. **(B)** Oil Red O staining was performed to evaluate the accumulation of lipid droplets in liver tissue. **(C)** HE staining to evaluate liver tissue damage. **(D**_**1 − 6**_**)**. Biochemical assays were conducted to determine the levels of AST, ALT, TC, LDL-C, TG, and HDL-C in tissues. **(E**_**1 − 3**_**)** The lactate concentration in serum and intestinal contents was measured using a lactate assay kit. **(F**_**1 − 3**_**)**. ELISA was used to measure the concentrations of inflammatory cytokines, such as IL-6, TNF-α, and IL-1β. *n* = 6. *P < 0.05, **P < 0.01, ***P < 0.001, ****P < 0.0001.

The results showed that the liver weight ratio of the NAFLD/CBX and NAFLD/ABX groups was significantly higher than that of the CBX group (Figure 2A, *P* < 0.05), accompanied by a marked increase in liver lipid droplet accumulation (Figure 2B). HE staining indicated that the NAFLD/CBX and NAFLD/ABX groups exhibited significant hepatocyte swelling, necrosis, and inflammatory cell infiltration, while the CBX group displayed normal hepatic cell structures (Figure 2C). Serum levels of AST, ALT, TC, LDL-C, and TG were significantly elevated in the NAFLD/CBX and NAFLD/ABX groups compared to the CBX group, while HDL-C levels were significantly reduced (Figure 2D, *P* < 0.05). Additionally, levels of IL-6, TNF-α, and IL-1β in liver tissue were significantly increased (Figure 2E, *P* < 0.05). Although the liver injury in the NAFLD/CBX group was slightly milder than in the NAFLD/ABX group, the difference between the two groups was not statistically significant (*P* > 0.05). These findings indicate that a HFD successfully induced NAFLD in the NAFLD/CBX and NAFLD/ABX groups, resulting in significant lipid metabolism disorders, inflammation, and liver injury.

To explore the role of Bif and its metabolic product, lactic acid, in the regulation of lipid metabolism disorders and inflammatory responses, antibiotic treatment (NAFLD/ABX group) was used to inhibit the endogenous gut microbiota in mice. Subsequently, NAFLD/ABX mice were administered either a *Bifidobacterium* suspension (NAFLD/ABX+Bif group) or glyceraldehyde-3-phosphate (NAFLD/ABX+Bif+G3P group) *via* gavage. The results demonstrated that, compared to the NAFLD/ABX group, the NAFLD/ABX+Bif group showed a significant reduction in liver weight ratio (*P* < 0.05), alleviation of liver lipid droplet accumulation, and pathological liver damage, with decreased hepatocyte swelling and necrosis (Figures 2A–C). Concurrently, serum levels of AST, ALT, TC, LDL-C, and TG were reduced, while HDL-C levels increased significantly (Figure 2D_1 − 6_, *P* < 0.05). Moreover, the concentrations of lactate in both serum and intestinal contents were significantly elevated (Figure 2E_1 − 3_, *P* < 0.05), and levels of pro-inflammatory factors (IL-6, TNF-α, and IL-1β) in the liver were markedly decreased (Figure 2F_1 − 3_, *P* < 0.05). When G3P was added (NAFLD/ABX+Bif+G3P group), lactate levels were further increased, and additional improvements in liver injury, reduced fat deposition, and decreased inflammatory responses were observed (*P* < 0.05). This suggests that Bif may alleviate liver injury and lipid metabolism disorders associated with NAFLD by promoting lactate production, which helps suppress inflammation. The addition of G3P likely enhances lactate production, further alleviating NAFLD symptoms. This enhanced effect may be due to G3P's ability to increase the metabolic flux toward lactate production, thus amplifying the beneficial effects of *Bifidobacterium* in regulating lipid metabolism and inflammation.

### 3.2 Exogenous lactic acid suppresses inflammatory response and NLRP3 expression in a NAFLD cell model

**Figure 3:**
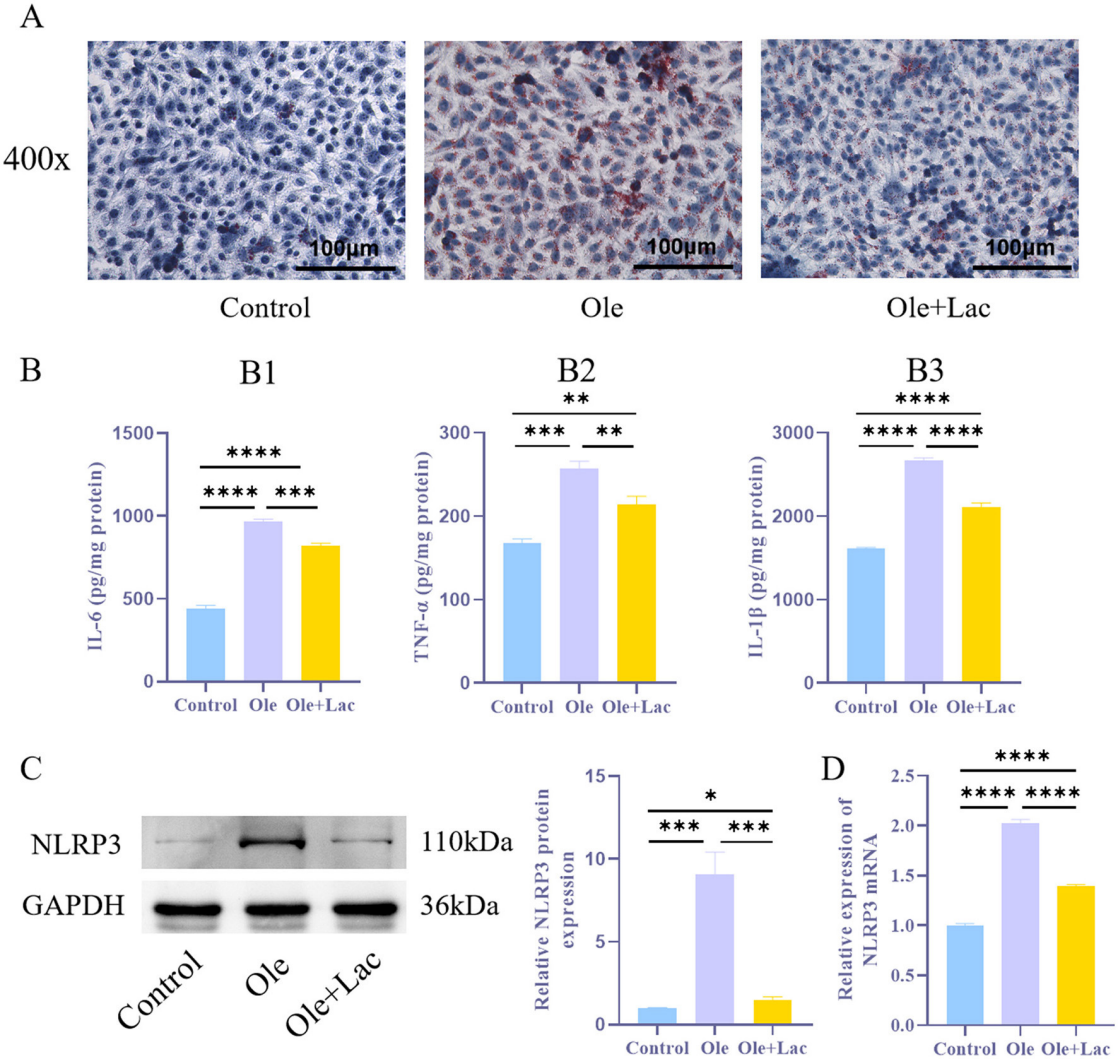
Exogenous lactic acid inhibits inflammatory responses in the NAFLD cell model. **(A)** Oil Red O staining to assess lipid droplet accumulation in cells. **(B**_**1 − 3**_**)**. ELISA analysis of inflammatory cytokines IL-6, TNF-α, and IL-1β levels. **(C, D)** WB and qRT-PCR analysis of NLRP3 protein levels in cells. *n* = 3. *P < 0.05, **P < 0.01, ***P < 0.001, ****P < 0.0001.

To investigate the role of lactic acid in alleviating lipid metabolism disorders, an *in vitro* NAFLD model was established using oleic acid-treated AML12 cells. The results indicated that, compared to the Control group, the Ole group exhibited a significant increase in intracellular lipid droplets (Figure 3A), accompanied by markedly elevated levels of IL-6, TNF-α, and IL-1β, as well as a significant upregulation of NLRP3 expression (Figures 3B–D, *P* < 0.05). In contrast, the Ole+Lac group demonstrated a notable decrease in intracellular lipid droplets, along with lower concentrations of inflammatory cytokines (IL-6, TNF-α, and IL-1β) and reduced NLRP3 expression (*P* < 0.05). These findings suggest that lactic acid effectively mitigates oleic acid-induced lipid accumulation and inflammatory responses while inhibiting NLRP3 inflammasome activation.

### 3.3 Exogenous lactic acid inhibits NLRP3 expression and alleviates inflammatory response and autophagy

**Figure 4:**
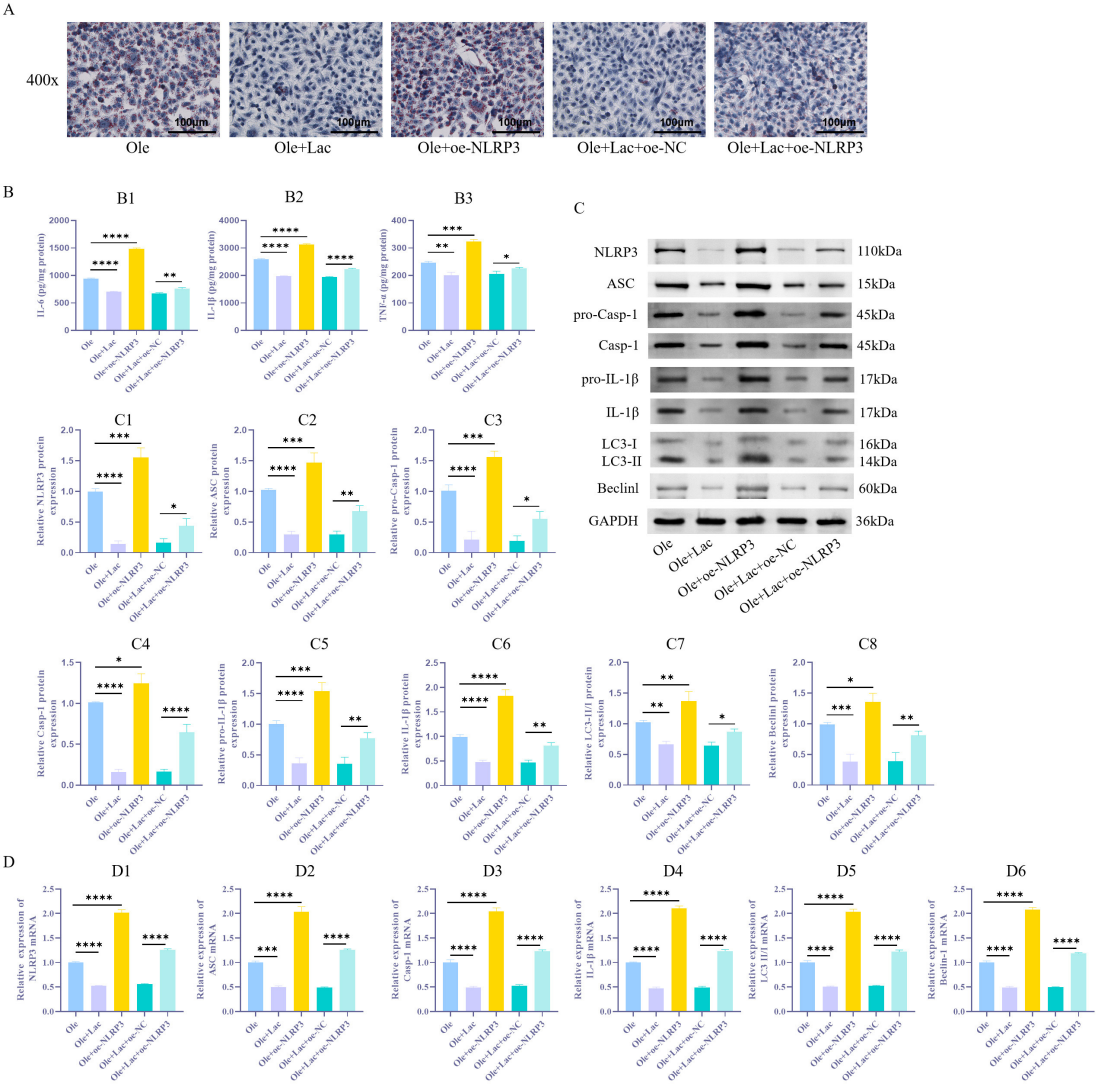
Exogenous lactic acid reduces autophagy in NAFLD cells and alleviates inflammatory responses induced by NLRP3 activation. **(A)** Detection of lipid droplet accumulation in cells *via* Oil Red O staining. **(B**_**1 − 3**_**)** ELISA measurement of inflammatory factors IL-6, TNF-α, and IL-1β levels. **(C**_**1 − 8**_**)** Protein expression levels of NLRP3 and autophagy-related markers (ASC, pro-Casp-1, Casp-1, LC3-II/I, Beclin-1, pro-IL-1β, and IL-1β) were assessed using WB in cells. **(D**_**1 − 6**_**)** qRT-PCR Detection of NLRP3, ASC, Casp-1, IL-1β, LC3, and Beclin-1 mRNA Expression Levels in Cells. *n* = 3. *P < 0.05, **P < 0.01, ***P < 0.001, ****P < 0.0001.

NLRP3 activation not only initiates inflammatory responses but also promotes autophagy to some extent. Earlier research has suggested that lactic acid may play a significant role in regulating autophagy. To verify whether lactic acid improves lipid metabolism by modulating the NLRP3 inflammasome and autophagy, this study conducted NLRP3 overexpression experiments in AML12 cells. The results showed that compared to the Ole group, the Ole+Lac group exhibited a reduction in lipid droplet (Figure 4A) s and a decrease in TNF-α, IL-6, and IL-1β levels (Figure 4B_1 − 3_, *P* < 0.05). Additionally, the expression of components related to the NLRP3 inflammasome (NLRP3, ASC, pro-Casp-1, and Casp-1), inflammatory factors (IL-1β and pro-IL-1β), and autophagy markers (Beclin-1 and LC3-II/I) was significantly reduced (Figures 4C_1 − 8_, 4D_1 − 6_, *P* < 0.05). In contrast, in the NLRP3 overexpression group (Ole+oe-NLRP3), all these indicators were significantly elevated (*P* < 0.05). Moreover, compared to the Ole+Lac+oe–NC group, the Ole+Lac+oe–NLRP3 group showed a significant increase in lipid accumulation and levels of inflammatory factors (IL-6, TNF-α, and IL-1β), along with marked upregulation of NLRP3 inflammasome and autophagy-related molecules (*P* < 0.05). The findings indicate that lactic acid may alleviate lipid metabolic disorders through the suppression of NLRP3 inflammasome activation and inhibition of autophagy, while NLRP3 overexpression reverses these protective effects of lactic acid.

### 3.4 The *Bifidobacterium* metabolite lactate improves the pathological condition of NAFLD mice by inhibiting NLRP3 expression and subsequently modulating inflammation and autophagy

**Figure 5:**
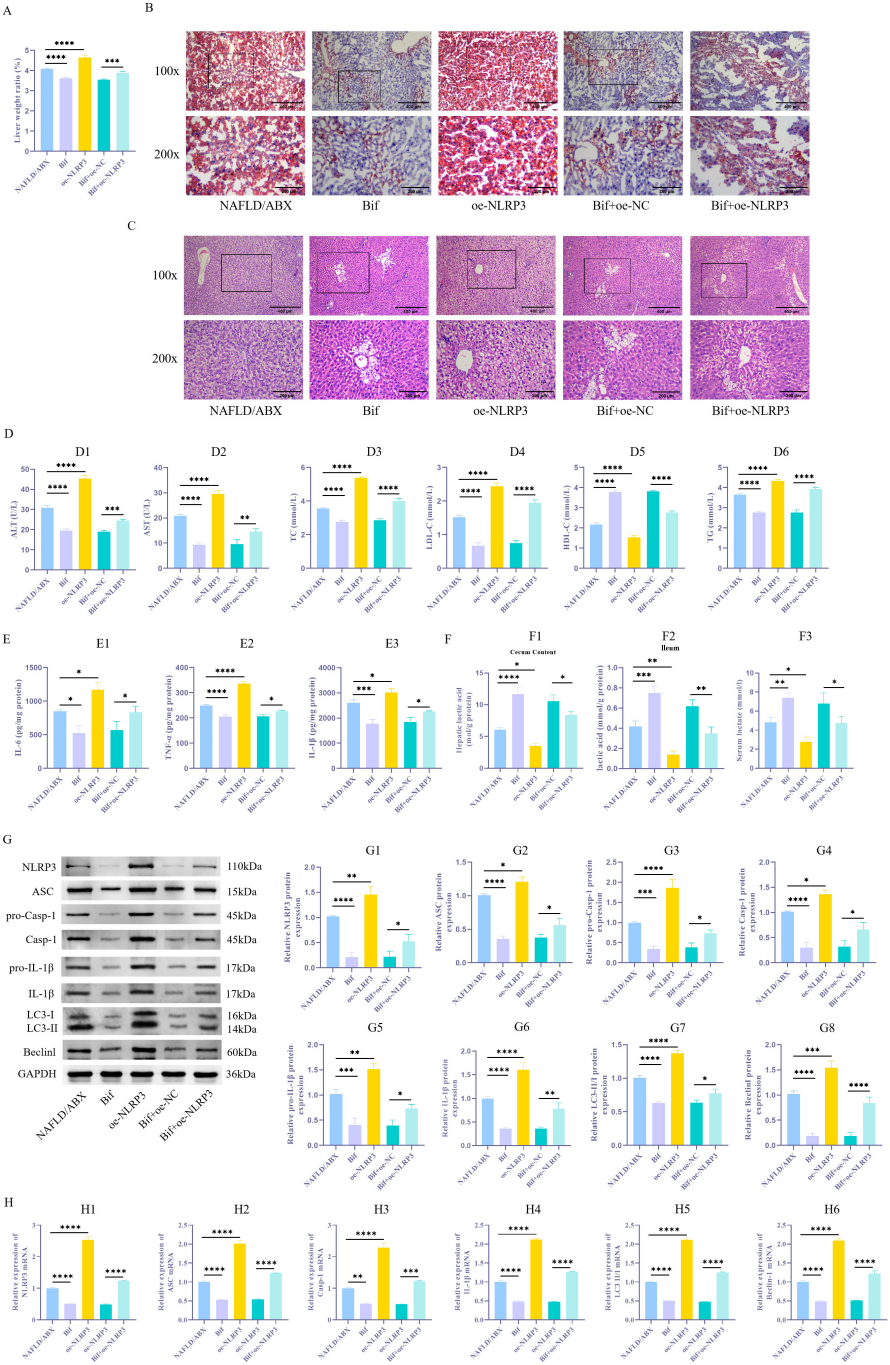
*Bifidobacterium* metabolite lactic acid improves inflammatory responses in NAFLD mice by inhibiting NLRP3-mediated autophagy. **(A)** Measurement of liver weight ratio. **(B)** Oil Red O staining was performed to evaluate lipid droplet buildup in liver tissue. **(C)** HE staining to evaluate liver tissue damage. **(D**_**1 − 6**_**)** Biochemical analysis of AST, ALT, TC, LDL-C, TG, and HDL-C levels in tissues. **(E**_**1 − 3**_**)** ELISA to determine levels of inflammatory factors IL-6, TNF-α, and IL-1β. **(F**_**1 − 3**_**)** The lactate assay kit was used to measure the concentration of lactic acid in the serum, as well as in the contents of the cecum and ileum. **(G**_**1 − 8**_**)** WB to examine NLRP3 levels and autophagy-associated proteins (ASC, pro-Casp-1, Casp-1, pro-IL-1β, IL-1β, LC3-II/I, and Beclin-1) in tissues. **(H**_**1 − 6**_**)** qRT-PCR Detection of ASC, Casp-1, IL-1β, LC3, and Beclin-1 mRNA Expression Levels in Cells. *n* = 6. *P < 0.05, **P < 0.01, ***P < 0.001, ****P < 0.0001.

To further validate the aforementioned mechanisms *in vivo*, we overexpressed NLRP3 in a NAFLD mouse model. The findings showed that relative to the NAFLD/ABX group, the Bif group displayed a notable reduction in liver weight ratio (*P* < 0.05), a marked reduction in lipid droplet accumulation, and alleviated hepatic tissue pathological damage, with less swelling and necrosis of hepatocytes (Figures 5A–C). Additionally, the concentrations of AST and ALT in the serum showed a significant decline, accompanied by marked reductions in TC, LDL-C, and TG, whereas HDL-C levels rose substantially (Figure 5D_1 − 6_, *P* < 0.05). The concentration of lactic acid was elevated, and levels of inflammatory factors IL-6, TNF-α, and IL-1β significantly decreased (Figures 5E_1 − 3_, 5F_1 − 3_, *P* < 0.05). Moreover, there was a substantial decrease in the levels of NLRP3, ASC, pro-Casp-1, Casp-1, pro-IL-1β, IL-1β, Beclin-1, and LC3-II/I (Figures 5G_1 − 8_, H_1 − 6_, *P* < 0.05). In contrast, mice in the oe-NLRP3 group exhibited exacerbated autophagy and inflammatory responses (*P* < 0.05). However, compared to the Bif+oe–NC group, mice in the Bif+oe–NC+oe–NLRP3 group showed an increased liver weight ratio, heightened lipid droplet accumulation, and aggravated hepatic tissue pathological damage. The levels of AST and ALT were elevated (*P* < 0.05), while TC, LDL-C, and TG levels increased and HDL-C levels decreased. Levels of IL-6, TNF-α, and IL-1β showed a significant increase, accompanied by higher expression of NLRP3, ASC, pro-Casp-1, Casp-1, pro-IL-1β, IL-1β, Beclin-1, and LC3-II/I (*P* < 0.05).

## 4 Discussion

NAFLD is a prevalent chronic liver condition characterized by excessive fat accumulation in hepatocytes, accompanied by persistent inflammatory responses (Engin, 2017). The pathological process of NAFLD is complex, involving various mechanisms such as dysregulated lipid metabolism and chronic inflammation (Wan et al., 2016). Among these, lipid metabolism disorder is regarded as a critical driving factor for NAFLD (Badmus et al., 2022). Due to the liver's inability to efficiently process and store fats, excessive lipids accumulate within hepatocytes, leading to inflammation and cellular damage (Chen et al., 2023). Without intervention, this condition may ultimately progress to liver fibrosis or cirrhosis, and even increase the risk of systemic metabolic syndrome, thereby raising the incidence of cardiovascular diseases (Lee et al., 2022). Therefore, in-depth research into the pathogenesis of NAFLD and the development of effective treatment strategies are of significant clinical importance.

In recent years, the gut-liver axis, a regulatory pathway through which the gut and liver interact *via* metabolism, immunity, and signaling pathways, has emerged as a critical factor in the onset and progression of NAFLD (Vallianou et al., 2021). The balance of gut microbiota is crucial for maintaining liver health. Disruption of the gut barrier and microbial imbalance can lead to harmful substances, such as endotoxins, passing through the portal vein into the liver, where they activate immune responses, inducing liver inflammation and metabolic disorders (Tilg et al., 2022). Consequently, restoring the normal function of the gut-liver axis through the regulation of gut microbiota and its metabolites have become potential therapeutic strategies for improving NAFLD (Li et al., 2024; Azad et al., 2018).

The NLRP3 inflammasome is a multi-protein complex that plays a key role in regulating host immune responses. In NAFLD, activation of the NLRP3 inflammasome is considered an important driver of liver inflammation and lipid metabolism disorders. The activation of NLRP3 inflammasome induces the release of pro-inflammatory cytokines such as IL-1β and IL-18, which exacerbate liver inflammation and cell damage, thus promoting the progression of NAFLD (Kanno et al., 2022). Inhibition of NLRP3 inflammasome activation has been shown to alleviate steatohepatitis (Jang et al., 2023), making the NLRP3 inflammasome a potential therapeutic target for NAFLD.

This study reveals, for the first time, the regulatory role of the bifidobacteria-derived metabolite lactic acid on the NLRP3 inflammasome in a NAFLD model. Lactic acid inhibits the activation of the NLRP3 inflammasome, alleviating chronic liver inflammation and improving lipid metabolism dysregulation. The inhibitory effect of lactic acid on the NLRP3 inflammasome may be mediated through multiple mechanisms: firstly, the acidic environment generated by lactic acid may directly affect the assembly of the NLRP3 inflammasome (Wan et al., 2016); secondly, lactic acid may modulate the gut microbiota and improve gut barrier function, thereby reducing endotoxin translocation and alleviating liver immune responses (Li et al., 2023, 2022).

Lactic acid not only exerts anti-inflammatory effects by inhibiting the NLRP3 inflammasome but may also improve lipid metabolism by regulating autophagy (Li et al., 2024; Azad et al., 2018). Autophagy is a key cellular degradation process that removes damaged organelles and proteins, thereby maintaining cellular homeostasis (Singh et al., 2009). In NAFLD, impaired autophagy is closely associated with fat accumulation, and lactic acid enhances autophagy activation, which helps reduce liver fat deposition (Watanabe-Yasuoka et al., 2023). Previous studies have indicated that lactic acid promotes lipid droplet degradation and fatty acid metabolism *via* autophagy, further supporting the findings of this study (Lee et al., 2018). This mechanism provides new evidence for the potential of lactic acid in NAFLD therapy.

In the mechanism of action of bifidobacteria and its metabolite lactic acid, the regulation of the gut-liver axis is particularly significant. Bifidobacteria ferment carbohydrates to produce lactic acid, which not only promotes its own growth but also lowers gut pH, optimizing the gut microbiota (Latif et al., 2023). As a short-chain fatty acid, lactic acid has multiple functions, including regulating gut pH, inhibiting pathogen growth, and modulating host immune responses (Cui et al., 2023). These functions help reduce the translocation of harmful substances from the gut to the liver, thereby alleviating liver inflammation and lipid metabolism disorders, further supporting the potential of lactic acid in NAFLD treatment. The synergistic effect between bifidobacteria and the gut microbiota should also not be overlooked. As a complex ecosystem, the overall collaborative function of the gut microbiota is vital for host health, suggesting that the therapeutic effect of a single bacterial species is only a part of the regulation of the gut-liver axis.

Lactic acid, as a metabolic product, not only plays an important role in regulating the gut-liver axis but also exerts multiple benefits by influencing autophagy and inhibiting the NLRP3 inflammasome. Therefore, the potential of lactic acid in NAFLD treatment extends beyond basic research, and its clinical application still requires further investigation. Existing studies suggest that lactic acid may need to be combined with specific formulations to optimize its therapeutic effects. For instance, improving the delivery method of lactic acid or combining it with other beneficial microorganisms may further enhance its therapeutic efficacy.

Although this study provides new insights into the treatment of NAFLD, it is still unclear whether lactic acid can be directly applied in clinical practice. Future studies should further explore the mechanisms of lactic acid in different types of liver diseases and investigate its clinical potential in metabolic diseases. Moreover, optimizing lactic acid formulations, drug delivery methods, and efficacy evaluation will be crucial in future research. Multi-center clinical trials assessing the safety and efficacy of lactic acid may provide more reliable scientific evidence for treating NAFLD and other metabolic diseases.

In conclusion, this study reveals the significant role of bifidobacteria and its metabolite lactic acid in improving NAFLD, particularly through the inhibition of the NLRP3 inflammasome and modulation of autophagy to alleviate liver inflammation and lipid metabolism disorders. This finding provides new potential targets and strategies for the prevention and treatment of NAFLD and offers important clinical translational potential.

## 5 Conclusion

The metabolite lactic acid produced by bifidobacteria alleviates inflammation and lipid metabolism disorders in NAFLD mice induced by a HFD through the inhibition of the NLRP3 inflammasome activation.

## Data Availability

The original contributions presented in the study are included in the article/Supplementary material, further inquiries can be directed to the corresponding author.
