# Supplementary material for: Exploring the alleviating effects of Bifidobacterium metabolite lactic acid on non-alcoholic steatohepatitis through the gut-liver axis
Source: Front Microbiol. 2025 Jan 7;15:1518150. doi: 10.3389/fmicb.2024.1518150 (PMC11756523; doi:10.3389/fmicb.2024.1518150)
Supplement: Supplementary file 1 [file Table_1.docx]

Figure 3C





GAPDH





NLRP3

Figure 4C





ASC





Beclin1


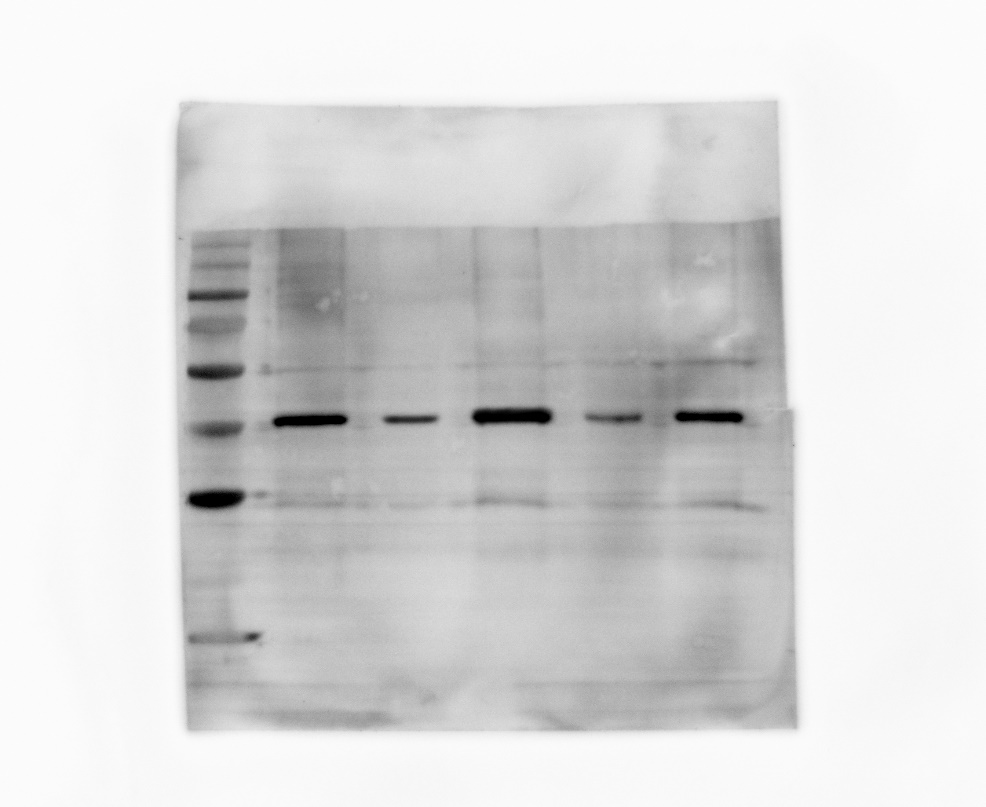


casp-1





GAPDH


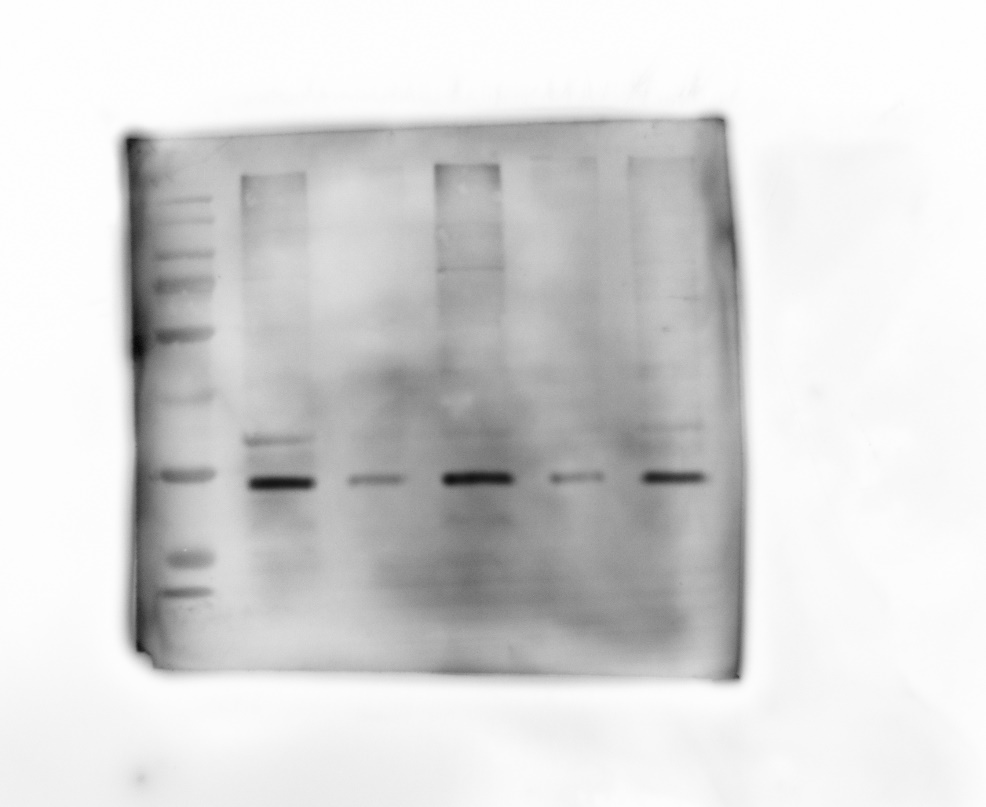


IL-1β


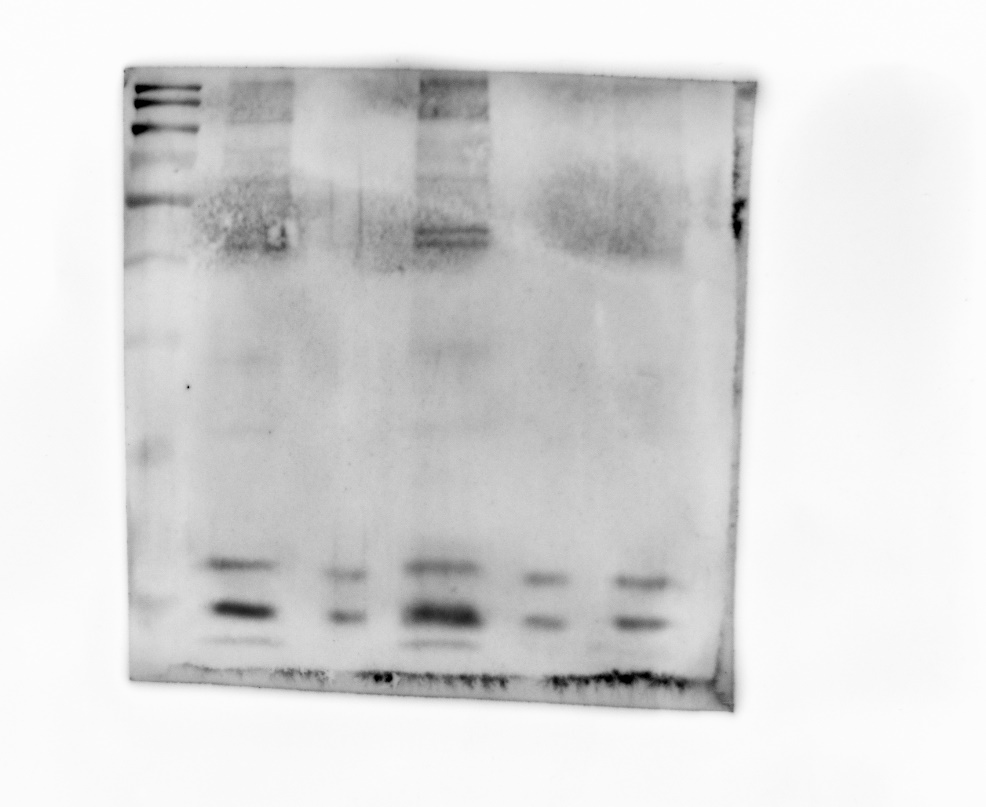


LC3-II&LC3





NLRP3


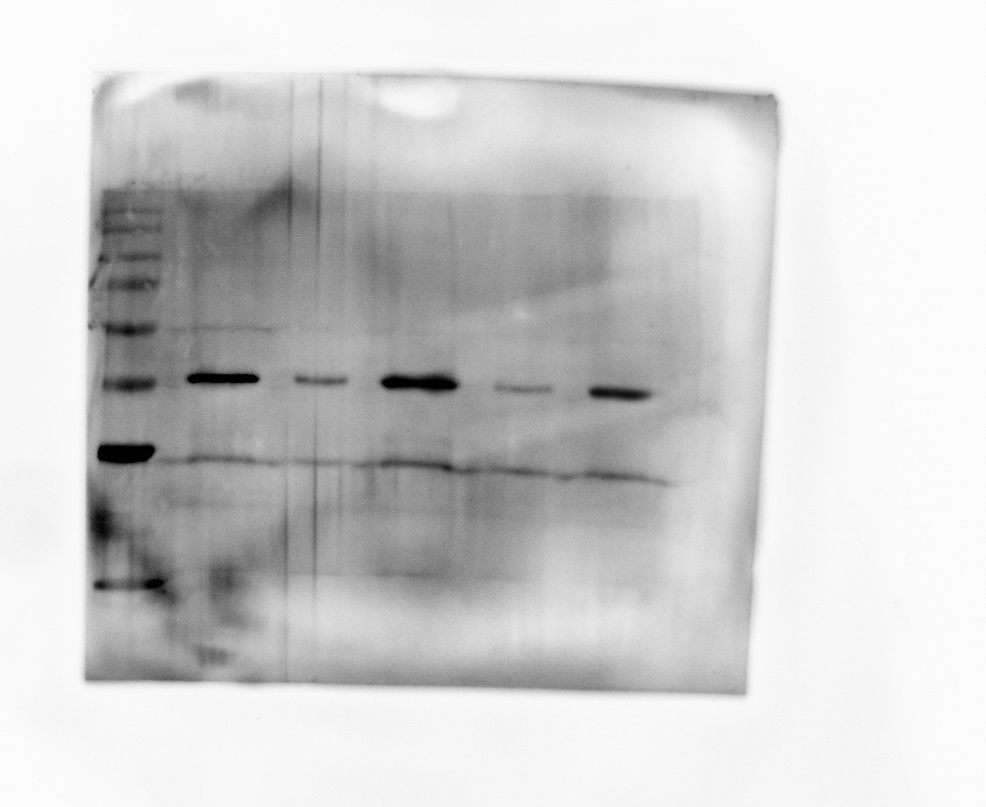


pro-casp-1


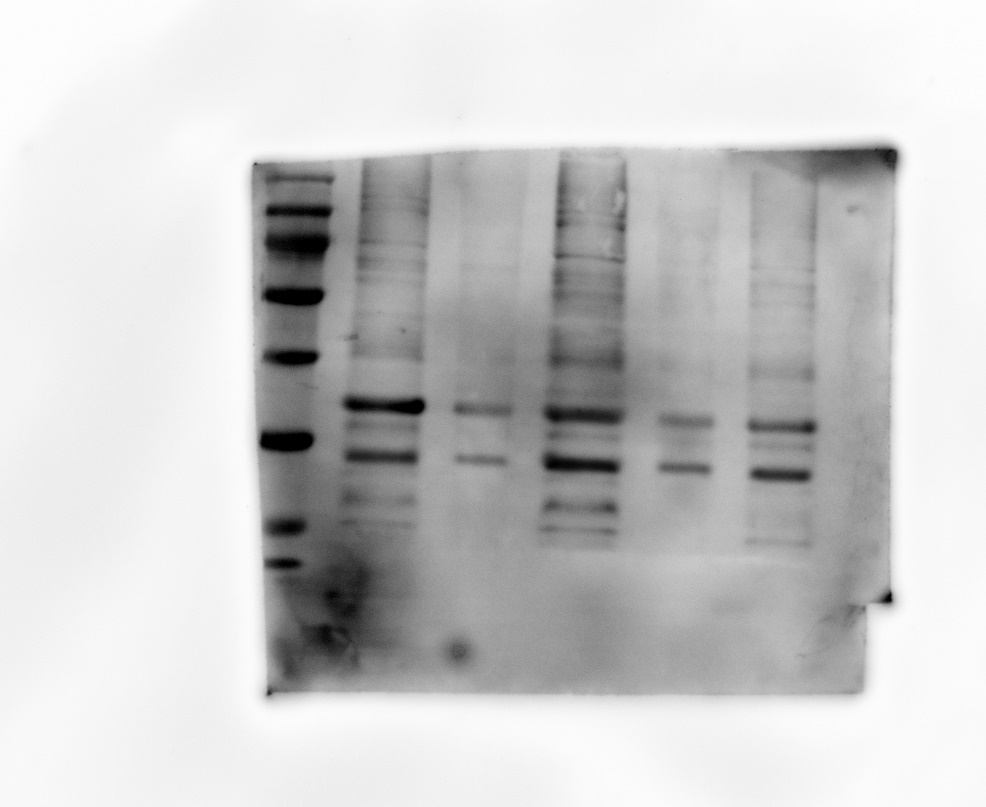


pro-IL-1β

Figure 5G





ASC


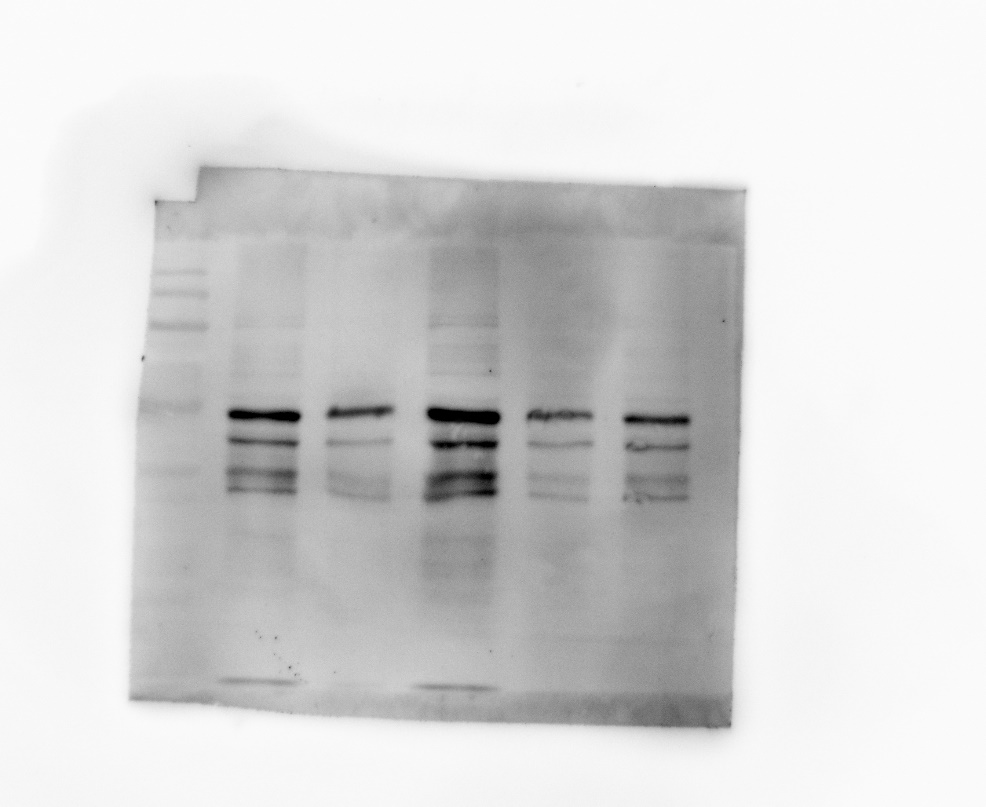


Beclin1


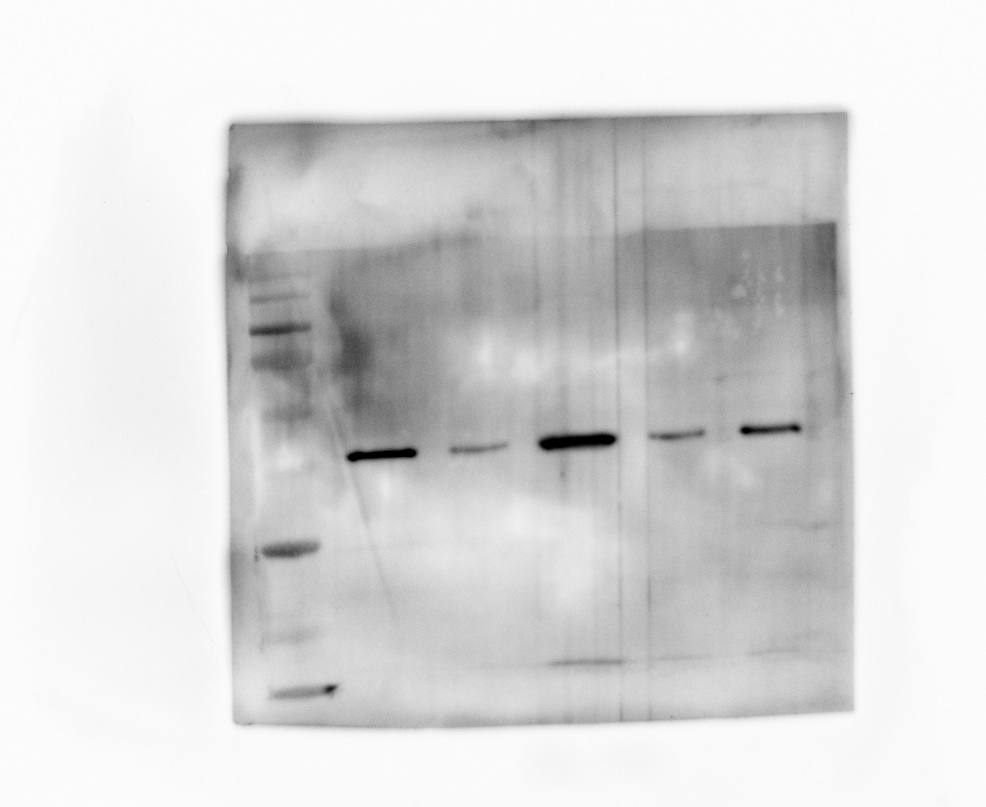


casp-1





GAPDH


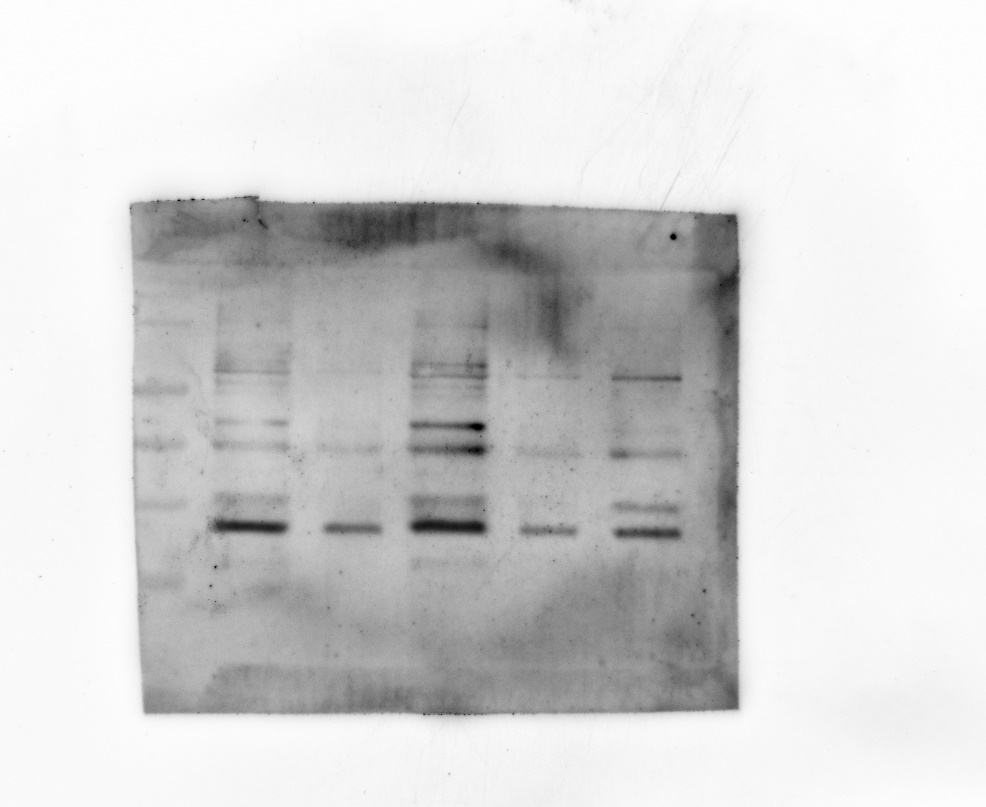


IL-1β


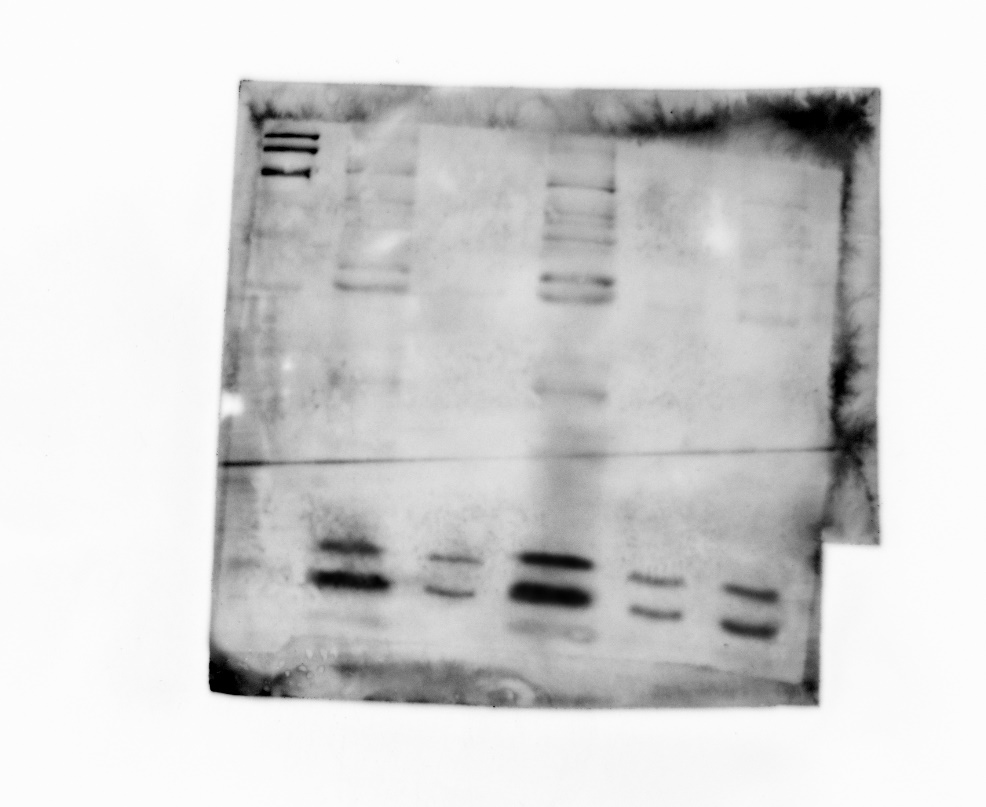


LC3-II&LC3





NLRP3


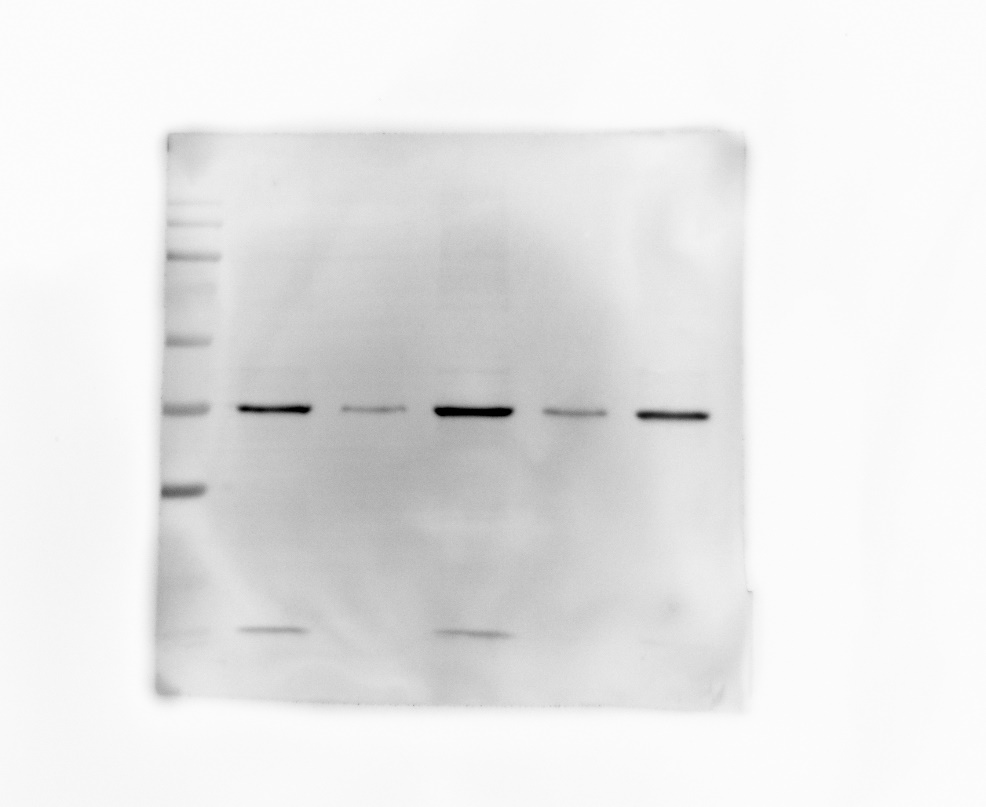


pro-casp-1


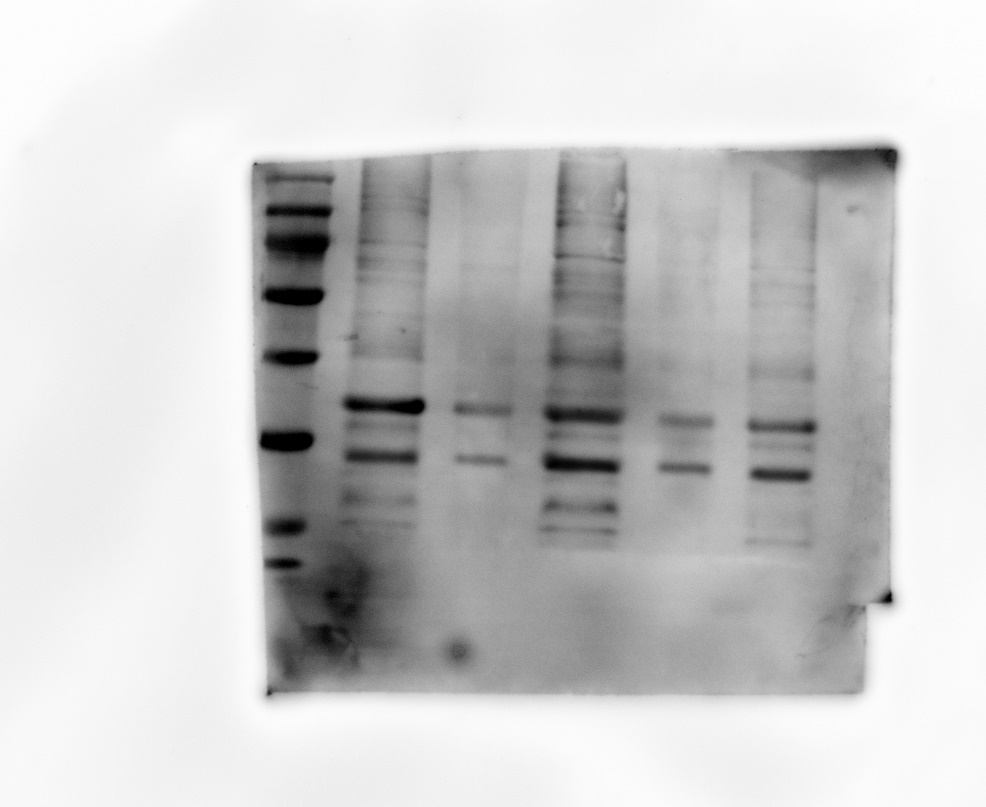


pro-IL-1β
